# Salmonella-Induced Immunothrombosis: An Unusual Case of Pulmonary Embolism in the Setting of Enteric Infection

**DOI:** 10.7759/cureus.94281

**Published:** 2025-10-10

**Authors:** Abeer Alsubaiei, Yousef Al-Shammari, Abdullah H Aljadi, Yousif A Ali, Saud Alzaid

**Affiliations:** 1 General Surgery, Sheikh Jaber Al-Ahmad Al-Sabah Hospital, Kuwait City, KWT; 2 Ophthalmology, Ministry of Health - Kuwait, Shuwaikh, KWT; 3 General Surgery, Al-Adan Hospital, Kuwait City, KWT; 4 General Surgery, Trinity College Dublin, Dublin, IRL; 5 General Surgery, Kuwait University, Kuwait City, KWT

**Keywords:** deep vein thrombosis (dvt), diabetic ketoacidosis (dka), immunothrombosis, non-typhoidal salmonella (nts), pulmonary embolism (pe), salmonella enteritis, venous thromboembolsim

## Abstract

Diabetic ketoacidosis (DKA) is a well-recognized metabolic emergency, often precipitated by infection or stress. Non-typhoidal *Salmonella* (NTS) gastroenteritis is usually self-limiting but can assume clinical significance in metabolically compromised patients, occasionally precipitating thrombotic complications. We describe a unique case where gastroenteritis in the setting of DKA was associated with pulmonary embolism (PE) in an ambulant patient. A 46-year-old previously healthy woman presented after a minor fall complicated by a seizure. Imaging revealed an incidental calcified right-frontal meningioma. Baseline investigations demonstrated new-onset diabetes with DKA and severe iron-deficiency anaemia, managed with insulin, fluids, and oral iron. On day seven, she collapsed with hypotension, tachycardia, and hypoxia. A Wells score of 6, right-ventricular dilation on bedside echocardiography, and markedly elevated D-dimer raised suspicion for PE, confirmed on CT pulmonary angiography. Anticoagulation with apixaban was commenced. Within 24 hours, she developed acute diarrhoea; stool cultures isolated NTS serogroup B. She received targeted ciprofloxacin with full clinical recovery. This case illustrates that acute *Salmonella* gastroenteritis in the metabolically stressed state of DKA can precipitate immunothrombosis and PE, even without systemic sepsis. It underscores the importance of timely recognition of PE in unexplained collapse and the need for integrated multidisciplinary management across neurological, endocrine, haematological, and infectious domains.

## Introduction

Non-typhoidal *Salmonella* (NTS) is a leading cause of foodborne gastroenteritis worldwide, responsible for an estimated 93 million cases and approximately 155,000 deaths each year [1]. Although most infections resolve without complications, around 1-5% progress to invasive disease, including bacteremia or vascular involvement [2]. Thromboembolic events, while relatively rare, are increasingly recognized as part of infection-associated immunothrombosis. Evidence from a nationwide Taiwanese cohort of 17,855 hospitalized NTS patients demonstrated nearly double the risk of deep vein thrombosis (DVT) and pulmonary embolism (PE) compared with matched controls (adjusted hazard ratios (aHRs) 1.83 and 1.84, respectively) [3]. Moreover, young adults aged 18-39 years showed the highest vulnerability, with risks rising nearly sixfold relative to their peers (aHR = 5.95 for DVT; 6.72 for PE) [1]

The association between *Salmonella* infection and venous thrombosis has been described for more than a century, although contemporary reports remain relatively sparse. Several case reports document NTS-related venous thrombosis or septic PE in otherwise low-risk individuals, including septic DVT, septic thrombophlebitis, and septic PE caused by various serotypes [3,4]. These observations, together with mechanistic data, support that infection itself can act as a potent prothrombotic trigger [5]. The underlying mechanism, often termed immunothrombosis, links infection-driven inflammation to intravascular coagulation [6].

Excess cytokine signaling, endothelial injury, tissue factor-dependent thrombin generation, and neutrophil extracellular traps (NETs) together create a strongly procoagulant milieu [7]. In murine* Salmonella* *typhimurium* infection, TLR4-dependent inflammation induces interferon-γ-mediated upregulation of podoplanin on monocytes/macrophages, which ligates platelet CLEC-2; genetic deletion or antibody blockade in this CLEC-2-podoplanin axis markedly reduces platelet-rich venous thrombosis [5]. Additional prothrombotic stressors such as diabetic ketoacidosis (DKA) can amplify risk via hemoconcentration, endothelial dysfunction, and inflammatory activation, and DKA has been associated with venous thromboses in observational series and case reports (including higher femoral CVC-associated DVT rates in pediatric DKA) [8].

In this case report, we aimed to present our case of a previously healthy middle-aged woman who developed acute PE in the setting of newly diagnosed DKA and subsequent non-typhoidal *Salmonella* gastroenteritis. We highlight how the combination of metabolic stress and infection may synergistically trigger immunothrombosis, underscoring the importance of recognizing infection-associated venous thromboembolism even in patients without traditional risk factors.

## Case presentation

A 46-year-old woman with no previous medical history presented after sustaining a minor fall in which she struck her forehead. Immediately afterward, she developed a brief generalized tonic-clonic seizure. A second seizure occurred in the emergency department and was terminated with 10 mg of intravenous diazepam. Post-ictally, she was confused and vomited twice but remained haemodynamically stable and afebrile. Non-contrast cranial CT revealed no acute injury but demonstrated a 0.9 × 0.9 cm calcified right-frontal extra-axial lesion, consistent with a small meningioma. She started on levetiracetam and was able to mobilize independently from day 2.

Baseline investigations uncovered newly diagnosed DKA (HbA1c >12% with metabolic acidosis) and severe microcytic anaemia (Hb 7.8 g/dL, ferritin 5 ng/mL, and transferrin saturation 2.5%). These were managed with intravenous insulin, fluids, and initiation of oral iron therapy.

**Figure 1:**
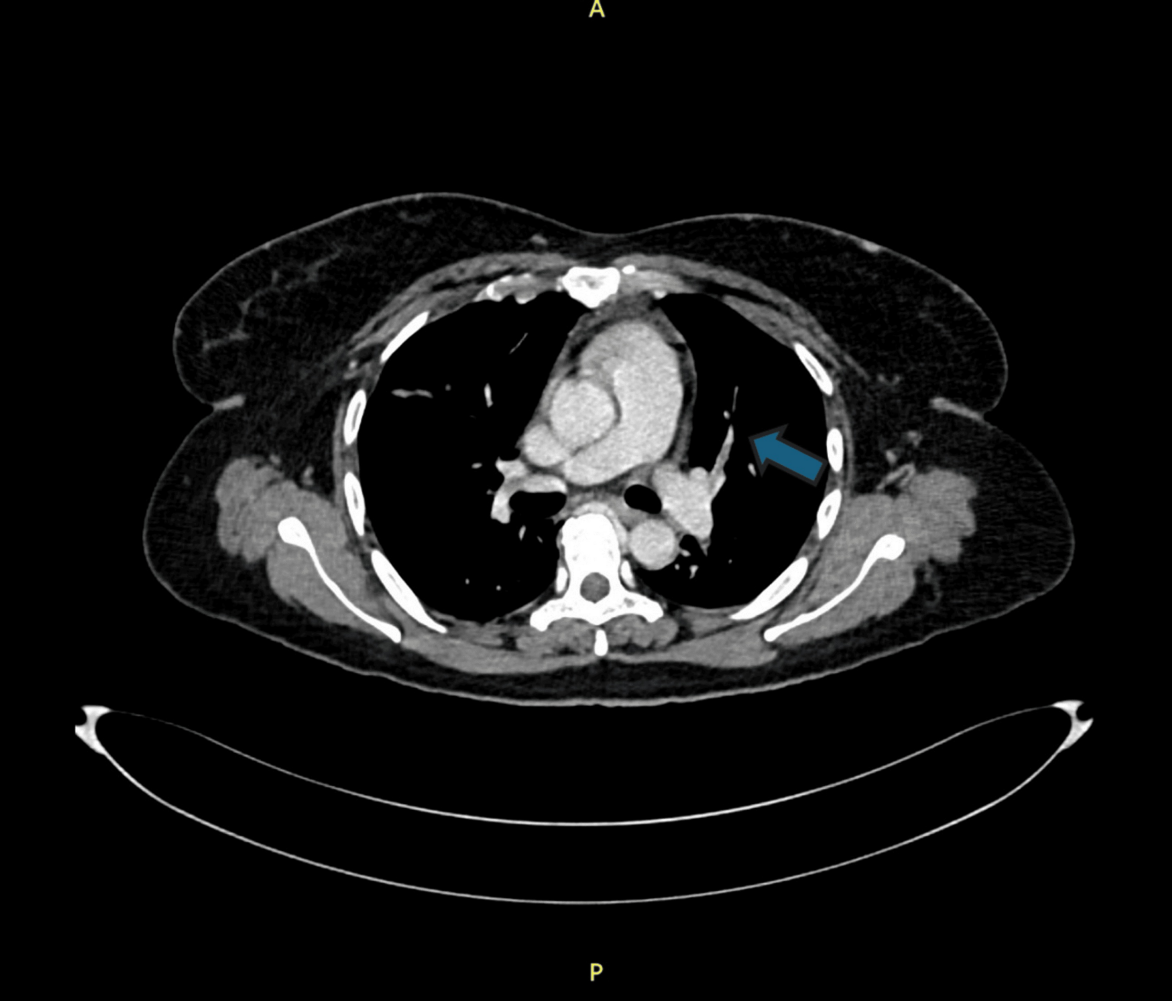
Hypodense filling defect seen involving left interlobar pulmonary artery, upper/anterior segmental and lower segmental branches denoting pulmonary embolism.

**Figure 2:**
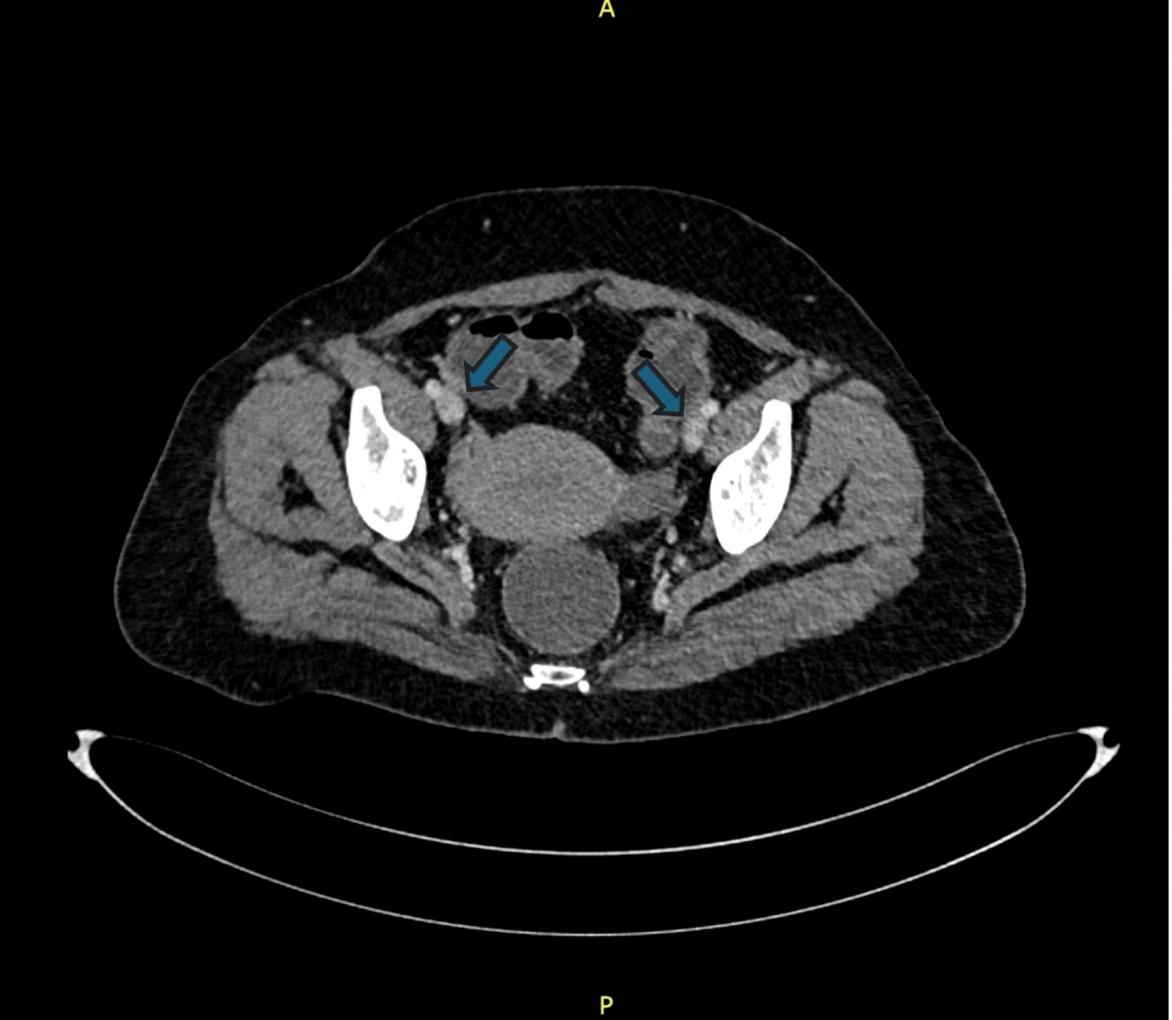
Bilateral external iliac veins show interluminal hypodense filling defects, more evident on the right. Thrombosis versus contrast acquisition artifact.

On day 7 of admission, the patient experienced sudden light-headedness followed by collapse while walking to the bathroom. She was found to be hypotensive (85/50 mmHg), tachycardic (120-130 bpm, sinus rhythm), and mildly hypoxic (SpO₂ 92% on room air). Her lungs were clear, and she remained alert. A Wells score of 6 (syncope, tachycardia, no alternative diagnosis more likely) and point-of-care echocardiography demonstrating a dilated right ventricle raised a strong suspicion of pulmonary embolism. This was supported by a markedly elevated D-dimer (>1330 ng/mL). CT pulmonary angiography confirmed segmental emboli in the left upper-lobe branches with filling defects in both external iliac veins, although bilateral lower-limb duplex ultrasonography was normal (Figures 1, 2).

Within 24 hours of this event, she developed acute gastroenteritis, passing five watery, cramp-associated stools. Stool culture grew non-typhoidal *Salmonella *serogroup B, while blood cultures were negative. She started on apixaban for pulmonary embolism, together with appropriate antibiotic therapy and supportive care.

Therapeutic anticoagulation was initiated with apixaban 10 mg twice daily for seven days, followed by 5 mg twice daily with a planned three-month course for provoked pulmonary embolism. Her renal and hepatic function were normal, meeting criteria for direct oral anticoagulant use. Repeat coagulation and renal panels were scheduled for six weeks, with thrombophilia testing (protein C/S, antithrombin, antiphospholipid antibodies) planned six weeks after cessation of apixaban. Pelvic CT venography did not confirm a discrete iliac clot, and an inferior vena cava filter was not indicated.

Empirical intravenous ciprofloxacin (400 mg q12h) and metronidazole (500 mg q8h) were started, with metronidazole discontinued on day 3 after stool-culture sensitivities confirmed ciprofloxacin susceptibility. She completed a 10-day oral ciprofloxacin course. Blood cultures remained sterile, and there were no features of systemic sepsis. In addition, she received food-safety counselling and vaccination advice (influenza and pneumococcal).

Her DKA resolved within 36 hours of intravenous insulin and fluid therapy. She was transitioned to a basal-bolus insulin regimen and later commenced on empagliflozin/metformin (Synjardy 12.5/1000 mg twice daily) once ketosis had cleared. She also received structured diabetes education, including sick-day rules. Iron-deficiency anaemia was treated with oral ferrous polymaltose, and follow-up was arranged with haematology to reassess iron indices.

The incidental calcified right-frontal meningioma required no acute intervention. Neurology scheduled surveillance MRI at six months.

She was discharged on day 11, haemodynamically stable, with scheduled follow-up in endocrinology, haematology, and neurology clinics. This case illustrates that acute Salmonella gastroenteritis, particularly in the metabolic stress of DKA, can precipitate immunothrombosis and pulmonary embolism even in ambulant patients. It underscores the importance of prompt imaging when unexplained collapse, tachycardia, hypoxia, and markedly elevated D-dimer are encountered.

## Discussion

Incidental intracranial meningiomas represent approximately 30% of new diagnoses, often discovered during imaging conducted for unrelated reasons [9]. Population studies estimate a prevalence of around 1.8% in older adults detected via MRI, with a marked female predominance, a pattern consistent with our patient [10]. Many such lesions remain asymptomatic, particularly when small and calcified, as in this case [11]. Differentiating etiologies of acute seizure in this context, trauma versus mass effect from a meningioma, poses a clinical challenge [12]. While minor head trauma can itself provoke a seizure, the presence of an incidental extra-axial lesion raises the possibility of a tumor-related epileptogenic focus. Although calcified meningiomas may be less metabolically active, development of seizures is reported in up to 10-70 % of patients with supratentorial non‑skull‑base meningiomas, depending on factors such as peritumoral edema and lesion location [13].

Management of such incidental meningiomas typically adheres to established consensus and guideline recommendations. For small, asymptomatic, presumed‑benign (WHO Grade I) lesions, an initial conservative approach with serial imaging, often referred to as “watch‑and‑wait," is endorsed over surgical intervention, unless growth or clinical symptoms emerge [14]. The European Association of Neuro‑Oncology (EANO) guidelines explicitly recommend observation as first‑line management for incidental, asymptomatic meningiomas, reserving resection for tumors that grow or become symptomatic [15].

Although DKA is classically linked with type 1 diabetes, up to one-quarter of adult patients presenting in DKA have newly diagnosed diabetes, and nearly one-third of DKA episodes occur in individuals with type 2 diabetes [16] DKA often follows an acute stressor, such as infection, or a precipitating illness, which elevates counter-regulatory hormones (glucagon, cortisol, growth hormone, and catecholamines), culminating in relative insulin deficiency and unchecked ketogenesis [17]. In illustrated cases, infections such as pancreatitis, trauma, or systemic illness have been well documented as triggers. In our patient, DKA occurred prior to the onset of acute *Salmonella *gastroenteritis; therefore, factors such as relative insulin deficiency and early systemic inflammatory response likely served as primary triggers, with gastroenteritis subsequently worsening the metabolic disturbance. The introduction of SGLT2 inhibitors post-DKA must be approached with caution [18,19]. While they confer cardiometabolic benefits, SGLT2 inhibitors carry a significantly heightened risk of both frank and euglycemic DKA. One study indicated a seven-fold increase in risk for DKA in type 2 diabetic patients taking SGLT2 inhibitors compared to other therapies [20].

This patient’s severe iron‑deficiency anemia (Hb 7.8 g/dL, ferritin 5 ng/mL, transferrin saturation 2.5 %) underscores the imperative to investigate both nutritional and occult bleeding as underlying causes. Iron‑deficiency anaemia is the most common global haematological disorder, and in adult men and post‑menopausal women, unexplained IDA merits urgent investigation due to its association with gastrointestinal pathologies, including malignancy, found in approximately one‑third of cases [21]. Clinically, severe anaemia may impair functional capacity, exacerbate fatigue, precipitate syncope, and hinder physiological recovery, particularly in acutely unwell individuals. In this patient, such compromise likely contributed to her collapse and hypotensive episode on day 7, underlining the broader systemic impact of profound anaemia.

In this patient, the diagnosis of PE was accurately triggered by clinical features, sudden light-headedness, hypotension, tachycardia, and mild hypoxia, culminating in a Wells score of 6, consistent with high pretest probability. Immediate point-of-care echocardiography revealing right ventricular dilation was pivotal in elevating suspicion, allowing expedited CT pulmonary angiography confirmation of segmental emboli. A large population-based cohort study reported that patients with NTS had nearly doubled risk of venous thromboembolism (adjusted subhazard ratio for PE 1.84, 95% CI 1.30-2.60), underscoring the pathogen’s prothrombotic potential even in the absence of systemic sepsis [3] Preclinical work similarly highlights how Salmonella-induced systemic inflammation triggers platelet activation, endothelial dysfunction, and delayed thrombosis, a mechanism referred to as thrombo-inflammation [22] The novelty of this case resides in the development of PE in an ambulant patient, without overt bacteremia or shock, suggesting that even localized enteric infection in the metabolically stressed state of DKA may precipitate thrombosis [23,24].

NTS typically causes an acute, self-limited diarrhoeal illness; however, metabolic and immune compromise, including diabetes and acute physiological stressors, raises the risk of severe or invasive disease. Classic and contemporary reviews document higher complication rates (e.g., bacteremia, extra-intestinal foci) in immunocompromised hosts, supporting the clinical significance of NTS in a patient concurrently recovering from DKA, even when blood cultures remain negative [25,26] In our patient, the episode of Salmonella gastroenteritis assumed clinical importance not because of systemic sepsis, but through its interaction with an already stressed metabolic environment. The acute diarrhoeal illness occurred shortly after the resolution of DKA, a state known to impair host immunity and increase susceptibility to infectious complications.

This case illustrates that acute* Salmonella* gastroenteritis in the metabolically stressed state of DKA may precipitate immunothrombosis and pulmonary embolism, even without systemic sepsis. It emphasizes the need for urgent evaluation of unexplained collapse, tachycardia, hypoxia, and markedly elevated D-dimer. The interplay of neurological, endocrine, haematological, infectious, and thromboembolic processes highlights the value of coordinated multidisciplinary management.

## Conclusions

In this ambulatory patient, new-onset DKA coincided with non-typhoidal *Salmonella* gastroenteritis and PE without bacteremia, highlighting infection-associated immunothrombosis as a plausible pathway linking metabolic stress and venous thromboembolism. Clinicians should maintain a low threshold for PE imaging when unexplained collapse, tachycardia, hypoxia, right-ventricular strain on bedside echocardiography, and markedly elevated D-dimer occur during metabolic decompensation. Conversely, the occurrence of PE in the context of acute gastrointestinal symptoms warrants targeted microbiological evaluation, as timely antimicrobial therapy may influence outcomes. In this case, therapeutic anticoagulation with apixaban, organism-directed ciprofloxacin, and coordinated multidisciplinary care (endocrinology, hematology, infectious diseases, neurology) led to full clinical recovery and provided a practical management template. The episode underscores the value of early diabetes education, careful post-DKA pharmacotherapy, and correction of iron-deficiency anemia to reduce physiological stress. We recommend heightened vigilance for venous thromboembolism in adults with DKA and intercurrent infection and encourage reporting of similar cases to refine risk estimates and management strategies.
